# AIDing cancer treatment: Reducing AID activity via HSP90 inhibition

**DOI:** 10.1002/eji.201545832

**Published:** 2015-07-07

**Authors:** Stefan Rebhandl, Roland Geisberger

**Affiliations:** ^1^ Laboratory for Immunological and Molecular Cancer Research3rd Medical Department with HematologyMedical OncologyHemostaseologyInfectiology and RheumatologyOncologic CenterParacelsus Medical UniversitySalzburgAustria; ^2^ Salzburg Cancer Research InstituteSalzburgAustria

**Keywords:** Activation induced deaminase, Antibody response, Class switch recombination, HSP90 inhibitors, Leukemia

## Abstract

The activation induced deaminase (AID) catalyses the two key events underlying humoral adaptive immunity: class switch recombination and somatic hypermutation of antibody genes in B lymphocytes. AID accomplishes this task by directly deaminating cytosines within the genomic immunoglobulin locus, thereby triggering a complex mutagenic process eventually leading to improved effector function of antibodies. However, it has long been noticed that AID can be aberrantly expressed in cancer and that its activity is not absolutely restricted to antibody genes, as substantial genome‐wide off‐target mutations have been observed, which contribute to tumorigenesis and clonal evolution of AID‐expressing malignancies. In this issue of the *European Journal of Immunology*, Montamat‐Sicotte et al. [*Eur. J. Immunol*. 2015. 45: 2365–2376] investigate the feasibility and efficacy of in vivo inhibition of AID with HSP90 inhibitors in a mouse model of B‐cell leukemia and in vitro with a human breast cancer cell line, thereby demonstrating that cancer patients may benefit from preventing noncanonical AID functions.

The activation induced deaminase (AID) was identified in 2000 as key enzyme for class switch recombination (CSR) and somatic hypermutation (SHM) in germinal center B cells 1, 2. By deaminating cytosines (C) within DNA of the antibody locus, AID generates uracils, which are excised from the DNA by the DNA repair machinery. During CSR and SHM, these uracils are replaced by random nucleotides by an error‐prone repair mechanism. If unrepaired, uracils base pair with adenine, leading to C>T transition mutations. These mutations result in a high diversity of antibodies, which are finally selected in the germinal center based on their affinity toward specific antigens during an immune response. In addition, AID‐dependent mutations lead to a substantial amount of double‐strand breaks within switch regions of antibody genes, thereby initiating CSR, the joining of distant constant regions of antibody genes (reviewed in 3).

As SHM and CSR are both highly mutagenic events, AID was soon suspected to also mediate off‐target DNA damage. Indeed, there is convincing evidence that AID‐dependent mutations also accumulate outside the antibody locus and that AID is responsible for a panel of chromosomal translocations as a by‐product of aberrant CSR 4, 5. Hence, AID off‐target damage has been shown to be involved in lymphomagenesis and clonal evolution of B‐cell malignancies 6, 7. Finally, AID was shown to be also expressed in non‐B‐cell tissue, particularly in many solid cancers, whereupon AID was also suggested to be a tumorigenic factor in stomach, breast, lung, liver, and colon cancers 8, 9, 10, 11, 12. In addition, an epigenetic function was attributed to the AID protein, as AID was shown to be capable of demethylating cytosines within promoter regions. AID was proposed to achieve this by deaminating methylated cytosines, thereby generating thymines. Hence, these thymines mismatch with guanines, which leads to the recruitment of DNA repair factors that eventually replace the thymine with a nonmethylated cytosine 13, 14.

To minimize off‐target effects, AID abundance and localization are tightly regulated (reviewed in 15). Normally, AID is excluded from the nucleus to avoid contact with genomic DNA and only a small fraction of AID molecules is transported into the nucleus from where it is subsequently shuttled back to the cytoplasm 16. Additionally, nuclear AID is very unstable, rapidly polyubiquitylated and degraded by the proteasome (Fig. 1) 17. In the cytoplasm, AID is quite stable as cytoplasmic AID is protected from proteasomal degradation by interaction with the heat shock protein HSP90 18. Consequently, inhibition of HSP90 by 17‐AAG leads to cytoplasmatic polyubiquitylation and degradation of AID (Fig. 1) 18.

**Figure 1:**
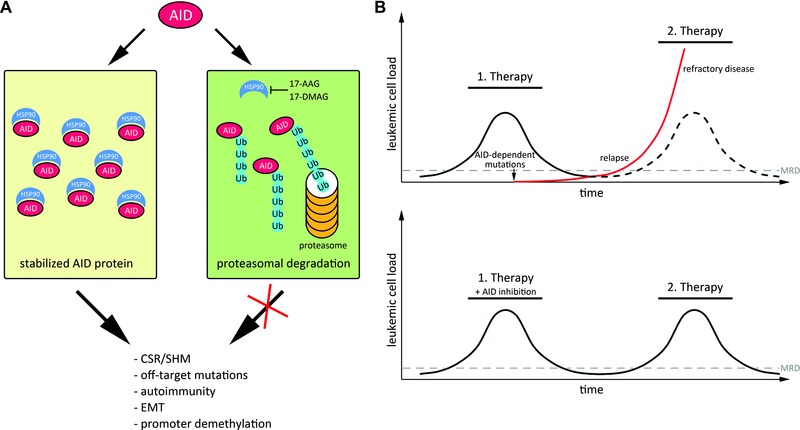
Model for AID stabilization and its activity during cancer progression. (A) AID is stabilized by interaction with HSP90 in the cytoplasm. Interference with HSP90, for example by the HSP90 inhibitors 17‐AAG or 17‐DMAG, leads to destabilization of AID, and its polyubiquitylation and proteasomal degradation. (B) Model for AID‐mediated clonal evolution of leukemic cells. During targeted therapy, the occurrence of resistant clones can be facilitated by AID‐dependent mutations, leading to relapse and refractory disease (top). The red line shows the occurrence of a treatment‐resistant cancer cell fraction. The ticked line indicates the unmutated cancer cell fraction, which remains sensitive to therapy. Simultaneous inhibition of AID could minimize the generation of subclonal mutations, which would confer therapy resistance (bottom). Abbreviations: AID: activation induced deaminase; HSP90: heat shock protein 90; 17‐AAG, 17‐DMAG: HSP90 inhibitors; Ub: ubiquitin; EMT: epithelial‐mesenchymal transition; CSR: class switch recombination; SHM: somatic hypermutation; MRD: minimal residual disease.

17‐AAG and its analog 17‐DMAG, two potent HSP90 inhibitors, have recently been introduced to cancer therapy and there are many ongoing clinical trials using these compounds as anti‐cancer drugs 19 (clinicaltrials.gov). HSP90 has been shown to stabilize a whole panel of cellular compounds such as intracellular receptors, kinases, and transcription factors and hence, many tumors rely on HSP90 for cell viability and proliferation 20, 21. Consequently, HSP90 inhibition represents a therapeutic strategy to reduce cell viability and proliferation in many cancers. However, it has not been evaluated whether 17‐AAG also interferes with AID function and whether part of its efficacy as an anti‐cancer drug may be attributed to an anti‐AID activity.

In this issue of the *European Journal of Immunology*, Montamat‐Sicotte et al. investigate the impact of 17‐AAG/17‐DMAG treatment on AID‐dependent functions in mice and a human breast cancer cell line 22. The authors show that, at the concentration used in their experiments, 17‐DMAG effectively diminishes AID‐dependent CSR and SHM in immunized mice in vivo, while B‐cell survival and proliferation remain unaffected. Furthermore, they demonstrate that in a mouse model for acute lymphoblastic leukemia, treatment of mice with 17‐DMAG delays tumor progression and strikingly, this effect is only significant in AID‐proficient tumors, indicating that the efficacy of 17‐DMAG is largely attributable specifically to AID inhibition. In a final experiment, the authors show that 17‐AAG treatment prevents AID‐dependent epithelial‐mesenchymal transition (EMT) in a breast cancer cell line, a process that leads to cancer progression and in which cells lose their epithelial features and gain mesenchymal properties, characterized by, for example, higher expression of the cytoskeletal filament Vimentin and the cell adhesion protein N‐cadherin 23. As EMT is most likely initiated by an AID‐mediated change in the gene expression profile, the authors also demonstrate that 17‐AAG impedes the noncanonical promoter demethylating function of AID.

AID is an established disease progression factor in many cancers. In this regard, AID may support clonal evolution by initiating subclonal mutations, which would eventually confer a growth advantage to the cell (Fig. 1) 7. Especially during therapy, tumors have to quickly adapt to a drug to overcome its anti‐cancer activity. Hence, AID may be exploited by the tumor to increase the spectrum of advantageous mutations, especially in response to novel targeted therapies where drugs interfere with specific molecules (molecular targets) involved in cancer cell growth and survival. Resistance to imatinib—a specific tyrosine kinase inhibitor—for example, is conferred by AID activity 24. Thus, there is a strong clinical relevance in inhibiting AID activity, in particular during targeted therapy. It is conceivable that an adjuvant AID inhibition (i.e. AID inhibition in addition to the respective targeted cancer therapy) may minimize clonal evolution of cancer (Fig. 1). Consequently, the efficacy of cancer treatment could be improved and the risk of generating refractory and relapsing disease could be decreased.

In summary, Montamat‐Sicotte et al. demonstrate that 17‐AAG/17‐DMAG, HSP90‐inhibiting drugs already used in clinical cancer trials, also lead to destabilization of AID, thereby preventing its detrimental activity in AID‐expressing cancers. Therefore, the use of HSP90 inhibitors as adjuvant drugs during targeted therapy of any AID‐expressing cancer should be considered as this may decrease relapse and improve remission rates.

## Conflict of interest

The authors declare no financial or commercial conflict of interest.

**Abbreviations**
AID: activation induced deaminase
CSR: class switch recombination
EMT: epithelial‐mesenchymal transition
SHM: somatic hypermutation
